# Inferring effective dynamics in large-scale networks of cortical neurons

**DOI:** 10.1186/1471-2202-14-S1-P266

**Published:** 2013-07-08

**Authors:** Netta Haroush, Shimon Marom, Paolo Del Giudice, Maurizio Mattia

**Affiliations:** 1 Faculty of Medicine, Technion - Israel Institute of Technology, Haifa, Israel; 2 Department of Technologies and Health, Istituto Superiore di Sanità, Rome, Italy; 3 Sezione Roma I, Istituto Nazionale di Fisica Nucleare, Roma, Italy

## 

Identifying a system entails determining effective nonlinear dynamics capable of quantitatively predict its behaviour in time and the repertoire of states under a suited choice of few key parameters [1]. System identification has been used to devise effective ways in controlling and improving the comprehension of both neuronal network and single cell dynamics [2,3]. Here, we extend a recently introduced theory-driven method for the identification of integrate-and-fire (IF) neuron networks [4], to characterize the spontaneous and evoked activity in large-scale networks of cortical neurons *in-vitro *[5]. The approach assumes that population bursts (PBs) observed in these biological preparations, result from the competitive interplay between a net excitatory synaptic reverberation driving the firing growth, and an activity-dependent inhibition akin to processes underlying spike-frequency adaptation (SFA) phenomena [2]. Mean-field approximation provides a theoretical framework [6] in which these two ingredients are quantitatively described by nonlinearly coupled differential equations for population firing rate and a fatigue variable, not experimentally accessible, determining the excitability level of the network. The fatigue state variable is reconstructed looking for an optimal anti-correlation between firing rates and fatigue level following electrical stimulations, which are periodically delivered to the network to elicit PBs. Inferred fatigue dynamics largely correlates both with failure rate in evoking PBs and with response delay between stimulation times and PB onsets. Nevertheless, spontaneously PBs, those that occur without exogenous stimulations, show a variability which is not fully captured by the identified two dimensional dynamics. Resorting to a principal component analysis, we further refined our system identification, and uncovered an additional hidden degree of freedom acting as an inhibitory force at the population level. This inhibition rapidly reacts to firing changes and expresses characteristic time scales of few tens of milliseconds, much shorter than the few seconds estimated for fatigue dynamics [7]. The composition of these slow and fast activity-dependent inhibitions finally allowed us to infer other dynamical features of the system such as the current-to-rate gain function of neurons involved in PBs.
